# Antibiotic consumption and its influence on the resistance in *Enterobacteriaceae*

**DOI:** 10.1186/1756-0500-7-454

**Published:** 2014-07-16

**Authors:** Miroslava Htoutou Sedláková, Karel Urbánek, Vladimíra Vojtová, Hana Suchánková, Peter Imwensi, Milan Kolář

**Affiliations:** 1 Department of Microbiology, Faculty of Medicine and Dentistry, Palacký University Olomouc, Hněvotínská 3, 775 15 Olomouc, Czech Republic; 2 Department of Pharmacology, University Hospital Olomouc, I. P. Pavlova 6, 77520 Olomouc, Czech Republic; 3 Department of Pharmacology, Faculty of Medicine and Dentistry, Palacký University Olomouc, Hněvotínská 3, 775 15 Olomouc, Czech Republic

**Keywords:** Antibiotics, Selection pressure, Resistance, *Enterobacteriaceae*

## Abstract

**Background:**

Increasing bacterial resistance to antibiotics is one of the most serious problems in current medicine. An important factor contributing to the growing prevalence of multiresistant bacteria is application of antibiotics. This study aimed at analyzing the development of resistance of *Enterobacteriaceae* to selected beta-lactam, fluoroquinolone and aminoglycoside antibiotics in the University Hospital Olomouc and assessing the effect of selection pressure of these antibiotics.

**Methods:**

For the period between 1 January 2000 and 31 December 2011, resistance of *Klebsiella pneumoniae, Escherichia coli, Enterobacter cloacae* and *Proteus mirabilis* to third- and fourth-generation cephalosporins, meropenem, piperacillin/tazobactam, fluoroquinolones and aminoglycosides was retrospectively studied. For the assessment of selection pressure of antibiotics, a parameter of defined daily dose in absolute annual consumption (DDDatb) based on the ATC/DDD classification and in relative annual consumption (RDDDatb) as the number of defined daily doses per 100 bed-days was used. The relationship between frequency of strains resistant to a particular antibiotic and antibiotic consumption was assessed by linear regression analysis using Spearman’s correlation. The level of statistical significance was set at p < 0.05.

**Results:**

A total of 113,027 isolates from the *Enterobacteriaceae* family were analyzed. There was a significant effect of selection pressure of the primary antibiotic in the following cases: piperacillin/tazobactam in *Klebsiella pneumoniae*, gentamicin in *Klebsiella pneumoniae* and *Escherichia coli* and amikacin in *Escherichia coli* and *Enterobacter cloacae*. Also, there was significant correlation between resistance to ceftazidime and consumption of piperacillin/tazobactam in *Klebsiella pneumoniae* and *Escherichia coli*. No relationship was found between consumption of third- and fourth-generation cephalosporins and resistance to ceftazidime or between fluoroquinolone consumption and resistance to ciprofloxacin.

**Conclusion:**

The study showed the effects of both direct and indirect selection pressure on increasing resistance to gentamicin, amikacin, piperacillin/tazobactam and ceftazidime. Given the fact that no correlation was found between resistance to fluoroquinolones and consumption of either primary or secondary antibiotics, we assume that the increasing resistance to fluoroquinolones is probably due to circulation of resistance genes in the bacterial population and that this resistance was not affected by reduced use of these antibiotics.

## Background

Gram-negative bacteria are currently the most frequent cause of nosocomial infections. Apart from *Pseudomonas aeruginosa*, these are mainly members of the *Enterobacteriaceae* family, most frequently *Klebsiella pneumoniae, Escherichia coli, Enterobacter* spp., *Proteus mirabilis* etc. In the University Hospital Olomouc (UHO; 1148 beds; 50,000 hospitalized patients per year), *Enterobacteriacae* are the most frequent pathogens causing nosocomial infections. For instance, they are responsible for 59% of nosocomial pneumonia cases [1]. Infections due to these bacteria are mostly treated with beta-lactam antibiotics, often conveniently combined with aminoglycosides and, in some cases, with fluoroquinolones. A significant problem in intensive care units is constantly increasing resistance to these antibiotics. It is known from earlier studies that bacterial resistance frequently results in inadequate antibiotic therapy, thus increasing morbidity and mortality rates and prolonging treatment and increasing treatment costs [2-4].

Resistance of Gram-negative bacteria to broad-spectrum penicillins and cephalosporins is currently so high that these important drug groups are very likely to lose their effectiveness. A 2006 study by Kolář et al. documented 39% prevalence of ESBL-positive strains of *Klebsiella pneumoniae* in intensive care units in the Czech Republic [5]. In 2011, the resistance of this species to third-generation cephalosporins in the Department of Anesthesiology and Intensive Care Medicine, UHO, reached 60% (unpublished data). Resistance to piperacillin/tazobactam was 63% and to fluoroquinolones even 67% (unpublished data). It is therefore necessary to analyze the development and the causes of resistance and to adjust administration of antibiotics to that trend.

The increase in bacterial resistance is contributed to by selection pressure of antibiotics, the clonal spread of multiresistant bacterial strains and recombination processes such as conjugation. Because of a different approach to preventing the spread of resistance it is important to differentiate whether the higher prevalence of multiresistant bacteria is caused by increased prescription of antibiotics or by clonal, horizontal spread. Selection pressure may be reduced by rational antibiotic policy and clonal spread of resistant strains may be reduced by adhering to hygiene and epidemiology principles. On the other hand, the transfer of mobile elements between bacterial cells cannot be significantly influenced in clinical practice.

This study aimed at analyzing the development of *Enterobacteriaceae* resistance to selected beta-lactam, fluoroquinolone and aminoglycoside antibiotics and particularly at assessing the effect of selection pressure of these antibiotics.

## Methods

### Group of bacterial strains

For the period between 1 January 2000 and 31 December 2011, a retrospective study was performed to assess the resistance of selected *Enterobacteriaceae* spp. (*Klebsiella pneumoniae, Escherichia coli, Enterobacter cloacae* and *Proteus mirabilis*) to selected beta-lactam, fluoroquinolone and aminoglycoside antibiotics. Bacterial strains were isolated from clinical samples (blood, urine, other body fluids and exudates, lower airway samples, needle biopsy samples, intravascular catheters, dialysate etc.) collected from UHO inpatients. Strains were selected from each patient in the following way: only one strain of a particular species was included which was isolated from a relevant sample as the first one over a time interval of 90 days. Identification was performed using standard microbiology procedures using the ENTEROtest 16 (Erba Lachema s.r.o.) and, in selected cases, the Phoenix automated system (Becton Dickinson) and MALDI-TOF (Biotyper Microflex, Bruker Daltonics).

The isolates were collected as part of standard patient care and no ethical approval was needed.

### Determining susceptibility to antimicrobial agents

Susceptibility to antibiotics was determined by the standard microdilution method according to the EUCAST criteria [6]. Reference strains *Escherichia coli* ATCC 25922, *Escherichia coli* ATCC 35218 and *Pseudomonas aeruginosa* ATCC 27853 were used for protocol quality control. Beta-lactamase phenotypes were determined by relevant phenotype tests and, if needed, the results were confirmed by detection of genes encoding the enzymes [7,8].

### Antibiotic consumption

Administration of antimicrobial agents and their selection pressure were assessed based on antibiotic consumption in the UHO between 2000 and 2011. The consumption data were obtained from the database of the hospital’s Department of Pharmacology. The utilization of individual antibiotics was expressed as absolute (total) annual consumption (DDDatb) in defined daily doses (DDD), based on the WHO ATC/DDD classification [9]. The relative annual consumption of antibiotics (RDDDatb) was determined as the number of defined daily doses per 100 bed-days (DBD) [10].

In order to assess selection pressure, association between resistance of the above enterobacteria to ceftazidime, piperacillin/tazobactam, ciprofloxacin, gentamicin and amikacin and consumption of relevant antibiotic groups (third- and fourth-generation cephalosporins, fluoroquinolones and aminoglycosides) and piperacillin/tazobactam was tested. Additionally, the relationship between the trends in resistance to the antibiotics and consumption of secondary antibiotics, i.e. drugs from other antibiotic groups than primary antibiotics from the same group, was assessed. The relationship between frequency of strains resistant to a particular antibiotic and antibiotic consumption was assessed by linear regression analysis using Spearman’s correlation. The level of statistical significance was set at p < 0.05.

## Results

Over the study period, a total of 113,027 isolates from the *Enterobacteriaceae* family (34,417 isolates of *Klebsiella pneumoniae*, 60,262 isolates of *Escherichia coli*, 3,624 isolates of *Enterobacter cloacae* and 14,724 isolates of *Proteus mirabilis*) were analyzed. The development of bacterial resistance to selected antibiotics is documented in Table 1. Resistance to third- and fourth-generation cephalosporins and piperacillin/tazobactam increased in all the studied species, with the exception of *Proteus mirabilis*. The increasing trend in resistance of *Klebsiella pneumoniae* appeared to be very serious. For instance, the resistance to ceftazidime increased from 5% in 2000 to 40% in 2011. In piperacillin/tazobactam, the increase was even greater; from 12% to 42%.

**Table 1:** **Development of *Enterobacteriaceae *** **resistance to selected antibiotics in the UHO in 2000–2011 (in percentages)**

		**2000**	**2001**	**2002**	**2003**	**2004**	**2005**	**2006**	**2007**	**2008**	**2009**	**2010**	**2011**
**CPR**	**KLPN**	18	22	19	14	14	16	11	20	42	39	41	41
	**ESCO**	4	4	2	2	2	3	4	10	11	12	12	11
	**ENCL**	15	18	22	21	24	15	15	22	30	23	35	32
	**PRMI**	4	6	2	2	2	2	2	2	2	2	2	2
**CTX**	**KLPN**	5	7	6	5	11	12	8	14	38	37	39	40
	**ESCO**	1	1	1	1	1	1	2	7	9	11	11	10
	**ENCL**	10	18	23	23	24	18	17	24	29	23	35	32
	**PRMI**	1	1	1	1	0	1	1	0	0	1	1	1
**CTZ**	**KLPN**	5	7	6	7	12	17	24	24	42	37	40	40
	**ESCO**	1	1	1	1	2	2	3	8	10	11	11	10
	**ENCL**	6	18	23	26	24	19	17	26	30	23	35	32
	**PRMI**	2	1	1	0	1	0	0	1	0	1	1	1
**CPM**	**KLPN**	NT	5	4	3	4	3	2	11	34	30	36	36
	**ESCO**	NT	1	0	0	1	0	1	6	8	10	10	9
	**ENCL**	NT	8	5	1	4	1	2	2	6	8	5	13
	**PRMI**	NT	1	1	0	0	1	0	0	0	1	0	0
**MER**	**KLPN**	0	0	0	0	1	1	0	0	0	0	0	1
	**ESCO**	0	0	0	0	0	0	1	1	0	0	0	0
	**ENCL**	0	3	0	2	0	2	1	1	1	1	0	0
	**PRMI**	0	0	1	0	0	0	1	0	0	0	0	0
**PPT**	**KLPN**	12	19	13	10	11	12	23	31	41	40	43	42
	**ESCO**	1	2	2	2	2	2	3	9	9	12	12	11
	**ENCL**	7	13	27	17	16	10	9	20	29	23	35	33
	**PRMI**	2	1	1	1	1	1	1	1	1	1	1	1
**OFL**	**KLPN**	6	8	15	10	10	11	29	28	30	36	38	41
	**ESCO**	3	5	5	8	10	13	15	19	22	21	23	21
	**ENCL**	3	6	5	2	3	4	3	6	7	6	15	10
	**PRMI**	11	14	14	17	19	17	22	29	33	34	35	33
**CIP**	**KLPN**	5	10	17	13	10	13	31	30	46	40	42	44
	**ESCO**	4	6	6	10	11	14	17	22	26	23	26	23
	**ENCL**	2	6	5	1	4	4	2	7	9	6	17	11
	**PRMI**	7	16	17	20	18	16	23	29	36	37	39	36
**GEN**	**KLPN**	12	15	17	17	19	17	17	18	35	32	37	37
	**ESCO**	3	4	4	4	5	7	6	8	9	8	8	7
	**ENCL**	15	5	0	4	2	4	1	5	8	4	6	15
	**PRMI**	48	39	30	23	22	21	18	17	19	21	20	20
**AMI**	**KLPN**	2	6	7	5	3	8	4	5	12	8	12	8
	**ESCO**	1	1	1	0	0	0	1	2	2	2	5	4
	**ENCL**	2	2	1	1	0	0	1	2	2	2	2	2
	**PRMI**	2	5	4	2	2	1	1	2	5	2	3	1

Legend: CPR - cefoperazone, CTX - cefotaxime, CTZ - ceftazidime, CPM - cefepime, MER - meropenem, PPT - piperacillin/tazobactam, OFL - ofloxacin, CIP - ciprofloxacin, GEN - gentamicin, AMI - amikacin, KLPN - *Klebsiella pneumoniae,* ESCO - *Escherichia coli,* ENCL *- Enterobacter cloacae,* PRMI - *Proteus mirabilis*, NT - not tested.

Over the same period, resistance to ciprofloxacin increased in all species, the highest being in *Klebsiella pneumoniae* (from 5% in 2000 to 44% in 2011) and *Proteus mirabilis* (from 7% in 2000 to 36% in 2011).

Resistance of *Klebsiella pneumoniae* and *Escherichia coli* to gentamicin gradually increased over the entire period (from 12% in 2000 to 37% in 2011 and from 3% in 2000 to 7% in 2011, respectively). In *Enterobacter cloacae*, the resistance initially decreased. However, from 2006, it increased, this being the same at the end as at the beginning of the study period, i.e. 15%. In *Proteus mirabilis*, the resistance even dropped from 48% in 2000 to 20% in 2011.

High susceptibility to meropenem and amikacin, i.e. 88-100%, remained unchanged in all the species throughout the entire period.

**Figure 1:**
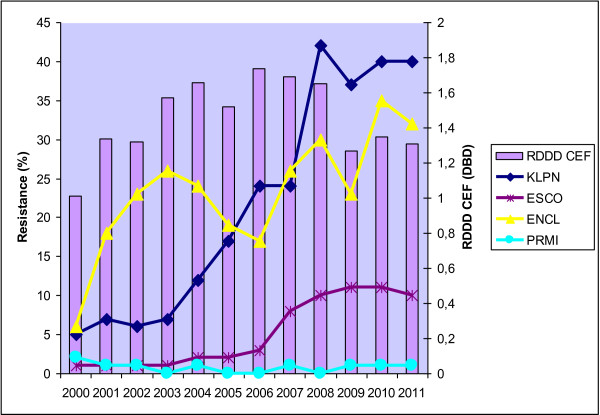
**Development of resistance of selected *Enterobacteriaceae *to ceftazidime with respect to consumption of third- and fourth-generation cephalosporins in the UHO in 2000 – 2011.** Legend: RDDD CEF – relative annual consumption of third- and fourth-generation cephalosporins in the UHO in 2000 – 2011, KLPN - *Klebsiella pneumoniae,* ESCO - *Escherichia coli,* ENCL *- Enterobacter cloacae*, PRMI - *Proteus mirabilis*.

**Figure 2:**
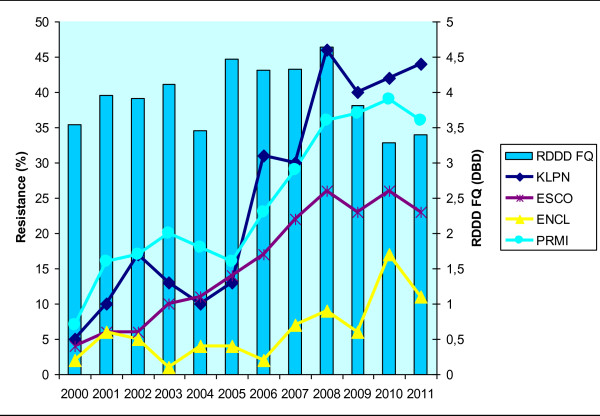
**Development of resistance of selected *Enterobacteriaceae *to ciprofloxacin with respect to consumption of fluoroquinolones in the UHO in 2000 – 2011.** Legend: RDDD FQ – relative annual consumption of fluoroquinolones in the UHO in 2000 – 2011.

**Figure 3:**
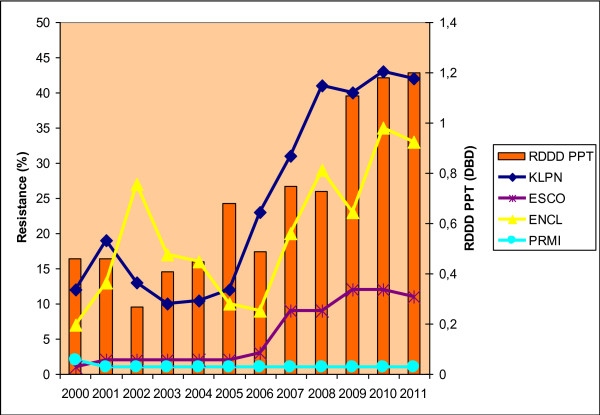
**Development of resistance of selected *Enterobacteriaceae *to piperacillin/tazobactam with respect to consumption of this antibiotic in the UHO in 2000 – 2011.** Legend: RDDD PPT – relative annual consumption of piperacillin/tazobactam in the UHO in 2000 – 2011.

**Figure 4:**
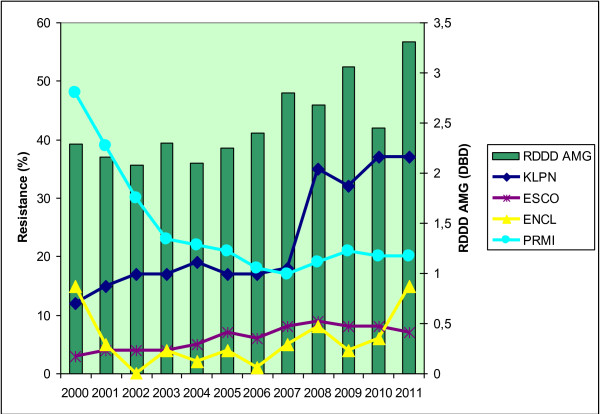
**Development of resistance of selected *Enterobacteriaceae *to gentamicin with respect to consumption of aminoglycosides in the UHO in 2000 – 2011.** Legend: RDDD AMG – relative annual consumption of aminoglycosides in the UHO in 2000 – 2011.

The relative annual consumption of the total amount of all antibiotics in the UHO rose by 21% between the years 2000 and 2011. The absolute and relative consumption rates of particular antibiotic groups are shown in Table 2. Correlation power, including statistical significance, is shown in Table 3. Statistically significant correlations were found between piperacillin/tazobactam consumption and resistance of *Klebsiella pneumoniae* (r = 0.85; p = 0.01) to this antibiotic, and between piperacillin/tazobactam consumption and resistance of *Klebsiella pneumoniae* (r = 0.84; p = 0.01) and *Escherichia coli* (r = 0.89; p = 0.01) to ceftazidime. There were also significant correlations between aminoglycoside consumption and resistance to gentamicin in *Escherichia coli* (r = 0.69; p = 0.02) and *Klebsiella pneumoniae* (r = 0.64; p = 0.03) and to amikacin in *Escherichia coli* (r = 0.74; p = 0.01) and *Enterobacter cloacae* (r = 0.63; p = 0.04). Non-significant positive correlations were shown in resistance of *Klebsiella pneumoniae* and *Escherichia coli* to the other antibiotics. Similarly, *Proteus mirabilis* was also found to have only statistically non-significant correlations.The correlations between antibiotic consumption and development of resistance are shown in Figures 1, 2, 3 and 4.

**Table 2:** Absolute annual consumption of selected antibiotics (DDDatb) in DDDs and relative annual consumption of antibiotics (RDDDatb) in DDD per 100 bed-days (DBD) at the UHO over the study period

		**2000**	**2001**	**2002**	**2003**	**2004**	**2005**	**2006**	**2007**	**2008**	**2009**	**2010**	**2011**
**Third- and fourth-generation cephalosporins**	DDDatb	3,725.5	4,925	4,742.4	6,065.9	6,447.3	5,504.6	6,019.45	5,981	5,886.13	4,417.88	4,640	4,148.25
	RDDDatb	1.01	1.34	1.32	1.57	1.66	1.52	1.74	1.69	1.65	1.27	1.35	1.31
**Piperacillin/tazobactam**	DDDatb	1,715.20	1,703.60	968.40	1,563.60	1,744.50	2,461.60	1,692.80	2,643.50	2,590.77	3,873.13	4,049.81	3,778.91
	RDDDatb	0.46	0.46	0.27	0.41	0.45	0.68	0.49	0.75	0.73	1.11	1.18	1.20
**Fluoroquinolones**	DDDatb	13,056.50	14,515.40	14,092.80	15,887.60	13,411.90	16,246.20	14,941.70	15,344.60	16,614.40	13,228.50	11,267.10	10,743.10
	RDDDatb	3.54	3.96	3.92	4.12	3.46	4.47	4.31	4.33	4.65	3.81	3.28	3.40
**Aminoglycosides**	DDDatb	8,455	7,928.9	7,468.4	8,879.3	8,121.7	8,183.6	8,338.1	9,915.50	9,555.92	10,625.54	8,418.23	10,437.27
	RDDDatb	2.29	2.16	2.08	2.3	2.1	2.25	2.4	2.8	2.68	3.06	2.45	3.31
**Total consumption of antibiotics**	DDDatb	171,316	172,463	142,822	161,919	146,848	164,857	167,993	184,238	186,943	187,945	192,398	177,030
	RDDDatb	46.39	47.05	39.73	41.97	37.92	45.38	48.45	51.97	52.34	54.1	55.99	56.09

**Table 3:** Results of Spearman’s correlation showing the relationship between frequency of strains resistant to a particular antibiotic and consumption of antibiotics potentially showing selection pressure

**Correlation 2000-2011**			**Statistical values**	
**Consumption of antibiotics**	**Bacterial resistance**		**R**	**p**
Fluoroquinolones	CIP	KLPN	0.0456	0.8797
		ESCO	0.0105	0.9721
		ENCL	-0.2355	0.4348
		PRMI	-0.1965	0.5146
Third- and fourth-generation cephalosporins	CTZ	KLPN	0.2285	0.4486
		ESCO	0.068	0.8216
		ENCL	0.1439	0.6333
		PRMI	-0.6139	0.0417
Third- and fourth-generation cephalosporins	PPT	KLPN	-0.1121	0.7101
		ESCO	0.0437	0.8848
		ENCL	-0.1329	0.6594
		PRMI	-0.4804	0.1111
Third- and fourth-generation cephalosporins	CIP	KLPN	0.1053	0.727
		ESCO	0.2285	0.4486
		ENCL	-0.1371	0.6494
		PRMI	0.1263	0.6753
Piperacillin/tazobactam	PPT	KLPN	0.8509	0.0048
		ESCO	0.8421	0.052
		ENCL	0.5009	0.0967
		PRMI	-0.175	0.5617
Piperacillin/tazobactam	CTZ	KLPN	0.8398	0.0053
		ESCO	0.8855	0.0033
		ENCL	0.4921	0.1027
		PRMI	0.0679	0.8218
Aminoglycosides	GEN	KLPN	0.6418	0.0333
		ESCO	0.6964	0.0209
		ENCL	0.523	0.0828
		PRMI	-0.6702	0.0262
Aminoglycosides	AMI	KLPN	0.4135	0.1703
		ESCO	0.7441	0.0136
		ENCL	0.6307	0.0364
		PRMI	-0.2708	0.369

R – Spearman’s rank correlation coefficient, p - statistical significance.

## Discussion and conclusions

Our work focused on the changing role of antibiotic selection pressure in the spread of bacterial resistance in *Enterobacteriaceae* in the 12-year period in the UHO. Although the total consumption of antibiotics increased during the study period, the use of antibiotics with the highest selection pressure potential such as third-generation cephalosporins and fluoroquinolones decreased. The growing use of antibiotics can be justified by a rising number of bacterial infections in the university hospital, i.e. a facility treating the most seriously ill patients at a higher risk of developing bacterial infections. On the other hand, lower consumption of third-generation cephalosporins and fluoroquinolones results from strict antibiotic policy implemented by the hospital’s antibiotic centre.

The presented results are indicative of a direct relationship between antibiotic consumption and resistance in piperacillin/tazobactam and aminoglycosides. Also suggested is indirect influence of selection pressure of secondary antibiotics in the case of piperacillin/tazobactam consumption and resistance to ceftazidime. Finally, in fluoroquinolones, no influence of selection pressure on the increasing trend of resistance was observed.

In the last two decades, several studies have been carried out on the relationship between consumption of antimicrobial agents and development of bacterial resistance, suggesting a positive correlation. Kolář et al. investigated the development of resistance in Gram-negative bacteria in 1994 – 1998 and assessed its association with consumption of various groups of antibiotics. They reported a statistically significant correlation between development of bacterial resistance and consumption of cephalosporins and fluoroquinolones [11]. A study by Urbánek et al. performed between 1997 and 2005 confirmed a positive correlation between the prevalence of ESBL-positive isolates of *Klebsiella pneumoniae* and consumption of third-generation cephalosporins [12]. These and other cases may document the widely accepted hypothesis of selection pressure and its impact on an increasing resistance to antimicrobial agents [13-15]. Our study showed a direct effect of selection pressure for increasing resistance only to gentamicin, amikacin and piperacillin/tazobactam. In a clear contrast to the aforementioned study of Kolář et al., resistance of *Enterobacteriaceae* to third- and fourth-generation cephalosporins and fluoroquinolones increased despite the lower use of these antibiotics. This implied correlation between the consumption of these two antibiotic groups and the bacterial resistance was not confirmed. Moreover, susceptibility of *Proteus mirabilis* to piperacillin/tazobactam and gentamicin was found to stagnate despite their increased use. It can be supposed that at present, bacterial resistance is not as directly dependent on the consumption of a particular antibiotic as shown by earlier studies.

Other, later studies showed an indirect relationship between consumption of a particular antibiotic and bacterial resistance to other antibiotics. A two-year study by Haller, performed in 2003 – 2004, showed a significant correlation between the use of second-generation cephalosporins and increasing prevalence of strains of *Enterobacter* spp. resistant to third-generation cephalosporins [16]. However, in the case of a direct relationship, i.e. the relationship between resistance to third-generation cephalosporins and/or piperacillin with tazobactam and their consumption, no significant correlation was found. Bosso et al. also investigated the association between antibiotic consumption and resistance in *Enterobacteriaceae*, finding that increasing or decreasing trends in resistance did not correlate with consumption of the primary antibiotic in any of the studied cases. However, dependence on consumption of secondary antibiotics was confirmed [17]. There were significant positive correlations between ciprofloxacin-resistant *Escherichia coli* and ceftriaxone consumption and between cefepime-resistant *Enterobacter cloacae* and piperacillin/tazobactam consumption. Bosso’s findings are consistent with the results of our study, suggesting that bacterial resistance may not be dependent on the use of a primary antibiotic; and decreased consumption of the primary antibiotics may not necessarily result in decreased resistance. Also Meyer et al., in their five-year interventional study, reported that after standard therapy of abdominal infections with third-generation cephalosporins had been changed to piperacillin/tazobactam combination, the resistance to third-generation cephalosporins did not decrease as it had been expected [18]. Meyer admitted the hypothesis that piperacillin/tazobactam combination may, through its selection pressure, increase the prevalence of *Enterobacteriaceae* producing broad-spectrum beta-lactamases, consistent with the finding in our study that administration of piperacillin/tazobactam correlated with the increasing trend of broad-spectrum cephalosporins resistance.

There are studies showing an interesting role of fluoroquinolones in antibiotic resistance. Similar to our study, a study by Spanish authors on the prevalence of *Enterobacteriaceae* resistant to broad-spectrum cephalosporins and fluoroquinolones in 1999 – 2010 and consumption of antibiotics in Spanish hospitals reported no correlation between the consumption of broad-spectrum cephalosporins and their resistance trends. However, the effect of fluoroquinolone administration on increasing resistance in both groups of antimicrobial agents was observed [19]. Our study found no relationship between the consumption and resistance in these two antibiotic groups. The resistance to ciprofloxacin rises progressively although the consumption of fluoroquinolones and broad-spectrum cephalosporins decreased years ago. Our results imply that selection pressure might be not an explanation for the increasing prevalence of fluoroquinolone-resistant *Enterobacteriaceae*.

From these facts, it may be assumed that the relationship between resistance and antibiotic administration is probably determined by additional factors and it cannot be influenced by a mere decrease in consumption. There are two other mechanisms of bacterial resistance spread besides the selective pressure: horizontal clonal spread of the identical multiresistant isolates and recombination processes such as conjugation of bacterial plasmids. In the UHO, the clonal spread represents a smaller proportion of resistant strains, as documented by some published papers [1,20,21]. After excluding the two above mechanisms, i.e. the selection pressure and the clonal spread of identical isolates, the increasing resistance rates in *Enterobacteriaceae* are thought to be caused by recombination mechanisms.

It is likely that the hypothesis of direct relationship between the total amount of antibiotics used and resistance to them is rather simplified given the relatively complicated ecological relationship between a bacterium and an antibiotic. Neither the defined daily doses nor the absolute annual consumption of antibiotics take into account important factors contributing to the development and spread of resistance, such as treatment adequacy, combination of antibiotics, proper dosage of drugs, adherence to treatment duration and intervals between individual doses. These factors possibly explain why the findings in several studies are very variable. Other, more precise methods should be sought to measure selection pressure and its influence on bacterial resistance.

A hypothesis has been published that asserts an imaginary threshold has been crossed and the resistance of *Enterobacteriaceae* to broad-spectrum antibiotics is maintained through transfer of mobile genetic elements encoding resistance [22]. That threshold is a certain level of resistance genes circulating in the bacterial population that are horizontally transmitted by recombination processes, causing the unstoppable spread of antibiotic resistance independent on their consumption. This hypothesis could be an explanation for the implication of our study that selection pressure does not play the main role in the increasing resistance to fluoroquinolones in *Enterobacteriaceae*.

It is likely that in antibiotic groups where the reasonable level of resistance has been exceeded, the increasing trend will be impossible to reverse. This leads to a heretical notion that rational antibiotic policy will neither reverse nor inhibit the increase of resistance and that this may actually be the end of the antibiotic era. Nevertheless, even this gloomy outlook should not discourage us from adhering to or improving the principles of rational antibiotic therapy as this is the only way of slowing this negative trend. To maintain the susceptibility of bacteria to at least those antibiotics that are still effective and to slow the increase of antibiotic resistance, it is necessary to continuously adhere to the principles of rational antibiotic policy and to try to define the determinants of bacterial resistance more clearly in order to introduce measures effectively preventing bacterial resistance from growing.

## Competing interests

The authors declare that they have no competing interests.

## Authors’ contribution

MK designed the study and co-ordinated the research, MHS was the principal investigator and takes primary responsibility for the paper; MHS, KU, VV and HS collected data; MK, MHS and PI wrote the paper. All authors read and approved the final manuscript.
